# Graphene Amination towards Its Grafting by Antibodies for Biosensing Applications

**DOI:** 10.3390/nano13111730

**Published:** 2023-05-25

**Authors:** Maxim K. Rabchinskii, Nadezhda A. Besedina, Maria Brzhezinskaya, Dina Yu. Stolyarova, Sergei A. Ryzhkov, Sviatoslav D. Saveliev, Grigorii A. Antonov, Marina V. Baidakova, Sergei I. Pavlov, Demid A. Kirilenko, Aleksandr V. Shvidchenko, Polina D. Cherviakova, Pavel N. Brunkov

**Affiliations:** 1 Ioffe Institute, Politekhnicheskaya St. 26, Saint Petersburg 194021, Russia; ryzhkov@mail.ioffe.ru (S.A.R.); sviatoslav.saveliev@gmail.com (S.D.S.); antonov@mail.ioffe.ru (G.A.A.); baidakova@mail.ioffe.ru (M.V.B.); pavlov_sergey@mail.ioffe.ru (S.I.P.); zumsisai@gmail.com (D.A.K.); alexshvidchenko@mail.ru (A.V.S.); pchervyakova@mail.ioffe.ru (P.D.C.); brunkov@mail.ioffe.ru (P.N.B.); 2 Department of Physics, Alferov University, 8/3 Khlopina Street, Saint Petersburg 194021, Russia; besedinadezhda@gmail.com; 3 Helmholtz-Zentrum Berlin für Materialien und Energie, Hahn-Meitner-Platz 1, 14109 Berlin, Germany; maria.brzhezinskaya@helmholtz-berlin.de; 4 NRC “Kurchatov Institute”, Akademika Kurchatova pl. 1, Moscow 123182, Russia; stolyarova.d@gmail.com

**Keywords:** 2*D* materials, aminated graphene, graphene modification, grafting, antibodies, photoelectron spectroscopy, biosensors

## Abstract

The facile synthesis of biografted 2*D* derivatives complemented by a nuanced understanding of their properties are keystones for advancements in biosensing technologies. Herein, we thoroughly examine the feasibility of aminated graphene as a platform for the covalent conjugation of monoclonal antibodies towards human IgG immunoglobulins. Applying core-level spectroscopy methods, namely X-ray photoelectron and absorption spectroscopies, we delve into the chemistry and its effect on the electronic structure of the aminated graphene prior to and after the immobilization of monoclonal antibodies. Furthermore, the alterations in the morphology of the graphene layers upon the applied derivatization protocols are assessed by electron microscopy techniques. Chemiresistive biosensors composed of the aerosol-deposited layers of the aminated graphene with the conjugated antibodies are fabricated and tested, demonstrating a selective response towards IgM immunoglobulins with a limit of detection as low as 10 pg/mL. Taken together, these findings advance and outline graphene derivatives’ application in biosensing as well as hint at the features of the alterations of graphene morphology and physics upon its functionalization and further covalent grafting by biomolecules.

## 1. Introduction

In the last decade, the derivatization of 2*D* crystals has arisen as one of the main trends in nanomaterial science, acting as a powerful tool for engineering crystals’ physics and achieving remarkable progress in the practical applications of these materials [1,2,3]. Owing to its versatile chemistry and ease of large-scale synthesis, graphene has become the most renowned 2*D* crystal in the field of derivatization with the appearance of a large family of chemically modified graphenes (CMGs) upon its covalent grafting by a diverse set of functional groups [3,4]. By altering the type and degree of functionalization, CMGs with the desired band structure, charge transport and electron field emission properties, optical characteristics and catalytic activity have been synthesized, advancing the development of next-generation graphene-based sensing systems, optoelectronic devices and metal-free catalysts [5,6,7].

Besides controlling physical properties, chemical derivatization also allows us to tune the wettability of graphene layers by various solvents and their dispersibility in polymer matrixes, as well as to covalently immobilize various biomolecules [8,9,10,11]. The latter feature is of particular interest within the field of biosensing applications of CMGs. Given their exceptional surface-to-volume ratio, the superb sensitivity of the electronic structure towards the appearance of any molecule, low intrinsic electrical noise and contact resistance, graphene films are beneficial in fabrication transducers for chemiresistive sensing platforms [12,13]. Capitalizing surface plasmon resonance effects in graphene derivatives yields another powerful approach to designing highly selective sensing devices [14,15,16].

However, the fundamental drawback is the total absence of selectivity in chemiresistive response towards any analyte [17], leveling all the advantages of graphene. To overcome this issue, biorecognizing molecules represented either by DNA strands, aptamers or antibodies can be introduced to the surface of graphene, granting it the desired selectivity [18,19,20,21,22]. Here the ease of functionalization comes out, allowing us not to rely on unstable non-covalent grafting by biomolecules through their Van-der-Waals interaction with the graphene layer [23,24] but to anchor them through covalent bonding. This offers the lower degradation of a sensor during its usage and enhances its sensitivity due to a higher impact of the charge transfer from the biorecognizing agent [11,23].

Numerous biosensing devices based on CMGs have been developed and tested up to date [10,11,18,19,20,21,22,25], proving that employing CMGs is an advantageous concept. Partially reduced graphene oxide (GO) and carboxylated (C-xy) graphene layers have mainly been trialed for this purpose with the covalent immobilization of DNA molecules and aptamers through covalent bonding with the hydroxyl and carboxyl groups present in these CMGs [10,11,18,20]. In turn, the use of aminated graphene is rarely reported and mainly limited by graphene layers being modified not directly by amines but by aliphatic linkers with the amine groups [22,26], which leads to disadvantages in the use of the graphene layer as a transducer. At the same time, the application of amine groups for the covalent immobilization through the carbo-amide bonding with the carboxyl fragments from the biorecognizing molecule would further expand the library of graphene-based biosensing devices, facilitating their performance and extending the range of the target analytes to be detected.

Herein, we consider a facile method for the GO conversion into aminated graphene through reductive amination and its subsequent covalent grafting by monoclonal antibodies towards IgM immunoglobulins. We report the chemistry, morphology and electronic structure of the acquired materials as well as the biosensing performance of the aminated graphene with the immobilized antibodies.

## 2. Materials and Methods

### 2.1. Materials

The aqueous dispersion of the initial GO employed for the synthesis of the aminated graphene was purchased from Graphene Technologies (Moscow, Russia, www.graphtechrus.com (accessed on 24 April 2023)). Formamide (CH_3_NO), 2-(N-morpholino)ethanesulfonic acid (MES free acid), 1-ethyl-3-carbodiimide hydrochloride (EDC), N-hydroxysuccinimide (NHS), Sulfo-NHS, phosphate buffered saline (PBS), Tween 20, sodium hydroxide (NaOH), sodium chloride (NaCl), glycine solution and hydrochloric acid (HCl) were acquired from Merck KGaA (Darmstadt, Germany).

Monoclonal antibodies towards human IgM immunoglobulins were purchased from ImteK, Ltd. (Moscow, Russia). IgM immunoglobulins from human serum (~95% HPLC, buffered aqueous solution) and bovine serum albumin (BSA) were purchased from Merck KGaA (Darmstadt, Germany).

All the organic solvents used in this work were acquired from Vecton Ltd. (Saint-Petersburg, Russia).

All the chemicals were of analytical purity grade, commercially available and used as received without additional purification.

### 2.2. Synthesis of rGO-Am and Pristine rGO

Aminated graphene was synthesized via liquid-phase reductive amination of the initial GO employing a modified Leucart reaction. In brief, 200 mL of 1 wt.% GO aqueous suspension was poured into a Teflon reaction vessel with the subsequent addition of 150 mL of CH_3_NO. The acquired reaction mixture was further heated at *T* = 165 °C for 48 h while stirring with a mixer at 250 rpm. Afterward, the suspension was cooled down to room temperature and the synthesized rGO-Am was copiously washed with deionized water (3 cycles) and isopropyl alcohol (3 cycles) using a glass filter with a pore size of 40 μm, resulting in the rGO-Am powder. To fabricate the rGO-Am films on the desired wafer and TEM grids for the subsequent examination by spectroscopic and microscopic techniques prior to and after immobilization of the antibodies, the fabricated rGO-Am powder was further dispensed in the isopropyl alcohol, achieving a concentration of 0.1 mg/mL with the subsequent deposition by the spray-coating technique described in Ref. [27].

The pristine rGO films were fabricated by drop-casting the GO aqueous suspension with a volume of 50–200 μL and concentration of 0.01 wt.% onto the Si wafers. These were subsequently dried overnight at room temperature (*T* = 25 °C) with subsequent annealing of the formed films at *T* = 650 °C in an ultra-high-vacuum chamber (*P* = 10^−9^ Torr) for 3 h:1.5 h to reach the indicated temperature, which was maintained for 1.5 h.

### 2.3. Grafting rGO-Am and rGO by Antibodies

To immobilize the monoclonal antibodies towards human IgG and IgM immunoglobulins, firstly MES buffer (125 mM MES, 0.9% NaCl, *pH* = 5.0) was prepared by suspending 2.44 g of MES free acid and 0.964 g of NaCl in 75 mL of deionized MilliQ water with the subsequent addition of 3.4 mL of 10N NaOH followed by final addition of deionized MilliQ water until a volume of 100 mL was reached. The *pH* values of the suspensions were evaluated with a Fisher Scientific Accumet Basic AB15 *pH* meter (Thermo Fisher Scientific, Waltham, MA, USA). Afterward, a stock solution was formed by dissolving 10 mg of EDC in 100 μL of the as-prepared MES buffer. Finally, reaction mixtures were prepared by further dilution of 1 μL of the EDC stock solution in 94 μL of the MES buffer with the subsequent addition of 5 μL of monoclonal antibodies solution with a concentration of 1 mg/mL via rigorous mixing by a vortex mixer for 2 min. Thus, acquired reaction mixture was left for 15 for the activation of antibodies by the EDC for the further covalent grafting of the graphene derivatives.

For the immobilization of the antibodies, substrates with the rGO and rGO-Am layers were placed into a Petri dish with a wetted cotton swab to maintain a humid environment, followed by deposition of 50 μL of the reaction mixture on their surface by mechanical pipette dispenser, ensuring the droplet completely covered the layer. Subsequently, the Petri dish was closed and the substrate with the reaction mixture was maintained for 2 h for the antibodies to immobilize on the surface of the treated CMG. After the indicated time, substrates were pulled out from the Petri dish and copiously washed by firstly placing them into the mixture of 100 mL of PBS and 100 μL of the Tween 20 detergent and then solely into 100 mL of PBS, with 30 min for each cycle, ensuring removal of all the physisorbed antibodies. Finally, the substrates with the CMG layers and immobilized antibodies were dried overnight at room temperature for further studies. Aminated graphene with the immobilized antibodies hereinafter is denoted as Am-ABd.

To assess the effect of the NHS and Sulfo-NHS on the efficiency of the antibodies’ immobilization, the same synthesis protocol was applied but with a reduction in the MES buffer volume to 93 μL and the addition of 1 μL of the NHS or Sulfo-NHS solution with concentrations of 0.6 mg/mL and 1.1 mg/mL, respectively.

### 2.4. Materials’ Characterization

The chemistry of the studied materials was examined via a set of core-level techniques and spectroscopic methods, namely XPS, XAS and Fourier transform infrared (FTIR) spectroscopy. FTIR spectra of the studied CMGs were collected from 1–2 μm thick films on the Si wafers, employing an Infralum-08 spectrometer (InfraLUM, St. Petersburg, Russia) operated in the attenuated total reflectance (ATR) mode. To acquire FTIR spectra of IgM antibodies, 10 μL of the corresponding solution with a concentration of 0.1 mg/mL was drop-casted directly onto the crystal of the spectrometer with the measurements performed before it dried off.

X-ray photoelectron and X-ray absorption spectra were collected at the ultra-high-vacuum experimental station of the Russian-German beamline of electron storage ring BESSY-II at Helmholtz-Zentrum Berlin (HZB) [28]. For the measurements, 50–100 nm thick films on the Si wafers were employed. Prior to the measurements, all the samples were evacuated down to a pressure of *P*~10^−9^ Torr for 24 h with no heating, still enough to remove all adsorbates but ensuring the retention of the materials’ chemistry. To consider the possibility of spatial heterogeneity, the spectra were collected from four equidistant spots of the sample with a size of ca. 200 × 100 µm. The difference between the obtained spectra was less than 3%, verifying the uniformity of all the samples, particularly the ones with the immobilized antibodies. Averaged spectra were employed for further processing.

The survey, C 1*s* and N 1*s* X-ray photoelectron spectra were measured with an excitation energy of 850 eV and an energy step of 0.5 eV for the survey ones, and 0.05 eV was used for the C 1*s* and N 1*s* ones. The collected X-ray photoelectron spectra were calibrated in accordance with the position of the reference Au 4*f*_7/2_ line lying at 84.0 eV. The C *K* and N *K* X-ray absorption spectra were acquired in the total electron yield mode by means of recording the sample drain current while altering the photon energy in the range of *hv* = 280–315 eV for the former one and *hv* = 395–420 eV for the latter one. The “magic” angle, α = 54.7°, was chosen for the XAS measurements to provide a nearly equal excitation of π- and σ-related states.

To process the acquired high-resolution C 1*s* and N 1*s* spectra, CasaXPS@ software (Version 2.3.16Dev52, Casa Software Ltd., Teignmouth, UK) was applied. Shirley background was chosen to fit all the spectra. Within the frame of C 1*s* deconvolution, a set of one asymmetric Doniach-Sunjic function (DS; 0.09–0.15; 90–250; GL90) and five symmetric Gaussian−Lorentzian functions with a ratio of 70–30% (GL(30)) were employed. For the N 1*s* deconvolution, four symmetric Gaussian−Lorentzian convoluted functions with a ratio of 70–30% (GL(30)) were applied. The as-recorded XAS spectra were conventionally normalized and smoothed following the algorithm described elsewhere [29].

The morphology of the synthesized materials was assessed by a set of microscopic techniques, namely scanning electron microscopy (SEM) and transmission electron microscopy (TEM) complemented by electron diffraction (ED) studies. The SEM images were collected with a JSM-7001F microscope (Jeol, Japan) from the Si wafers covered by arrays of individual flakes of GO, rGO-Am and Am-ABd deposited by drop-casting 25 μL of corresponding 0.01 wt.% suspension. TEM images and ED patterns were acquired from individual platelets of GO, rGO-Am and Am-ABd deposited from the aqueous and isopropyl suspensions with a concentration of 5 × 10^−5^ wt.% on the surface of TEM Cu grids (400 Mesh) with the use of a Jeol JEM-2100F microscope (Jeol, Ltd., Tokyo, Japan).

The size distribution of the rGO-Am layers was estimated by means of the laser diffraction (LD) method with the corresponding 0.05 wt.% isopropyl suspension measured with a Mastersizer 2000 (Malvern Panalytical, Malvern, UK). The complex refractive index for GO was set as 2.3 + 0.01i according to published data [30], and the Fraunhofer model was chosen for the scattering pattern processing as the most appropriate for the two-dimensional CMG platelets [31].

To examine the electronic structure of the studied materials, the X-ray photoelectron valence band (VB) spectra and the secondary electron (SE) cut-off spectra with an excitation energy of *hv* = 130 eV and an energy step of 0.05 eV were measured. With this particular excitation energy, the cross-sections of 2*p* and 2*s* states are almost equal, allowing us to precisely derive the effect of these states on the structure of the materials’ VB. To provide a convenient comparison and to eliminate stochastic noise, all the spectra were accurately smoothed and normalized to have an equal intensity of the dip between the O 2*s* peak and higher spectral features.

Processed SE cut-off spectra were used to calculate the work function values of the materials under study using the following relationship:

(1) $${e\Phi }_{m}=hv-(E_{F}-E_{SEC})$$

where *hν* = 130 eV is the photons’ energy, and *E_F_* and *E_SEC_* are the positions of the Fermi level and cut-off threshold, which are both represented in the kinetic energy scale [32].

For the biosensing studies, the immobilization of the antibodies was carried out on the two-electrode chips composed of the 100 nm thick rGO-Am film spray-coated on the quartz substrates (75 × 30 mm in size) equipped with pair of Au electrodes distanced by 25 mm. To examine the sensing performance of the fabricated biosensing chips, PBS solutions of the human IgM immunoglobulins with concentrations in the range of 10–500 pg/mol and a volume of 50 μL were deposited by a mechanical dispenser that simultaneously measured the resistance of the layer. Purification of the chips’ surface was performed by a constant flow of the GHCl detergent at a rate of 1 mL/s for 45 s with subsequent drying by airflow of 100 sccm for 2 min. GHCl detergent was prepared by mixing 0.1 M glycine solution with HCl to reach a *pH* of 2.5–3. The resistance measurements were carried out via Keithley 6487 picoammeter/voltage source (Keithley Instruments, Cleveland, OH, USA). The chemiresistive response was calculated as the relative change in resistance as a percentage:

(2) $$S=\frac{R_{IgM}-R_{PBS}}{R_{PBS}}\cdot 100\%$$

where *R_PBS_* is the film resistance being covered by PBS stock solution, and *R_IgM_* is the film resistance upon being exposed to the PBS solution of the IgM immunoglobulins from human serum. To verify the predominant role of the immobilized antibodies in detecting immunoglobulins and evaluate the selectivity of the detection, chemiresistive responses of a pristine rGO-Am film towards the 500 pg/mol solution of immunoglobulins in PBS and of Am-ABd film towards PBS solution mixed with the BSA albumin were additionally collected.

## 3. Results

### 3.1. Chemistry of the Derived CMGs

**Figure 1:**
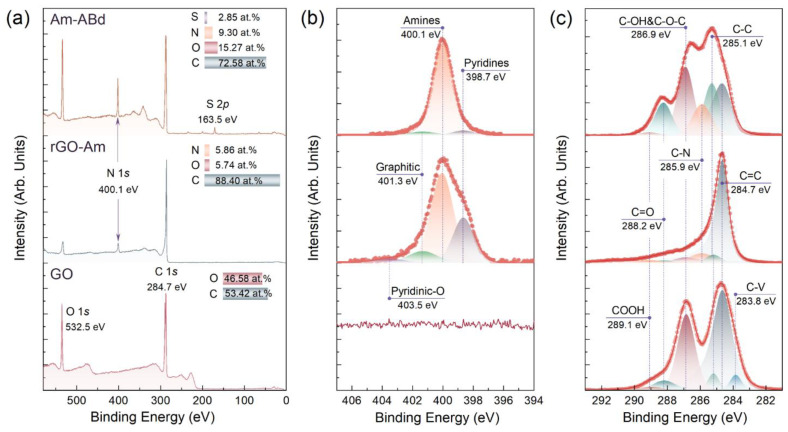
Examination of the CMGs’ chemistry by means of X-ray photoelectron spectroscopy. (**a**) The survey; (**b**) high-resolution N 1*s;* and (**c**) C 1*s* spectra of the initial GO, rGO-Am and Am-ABd layers.

Figure 1a displays the X-ray photoelectron survey spectra of the studied materials. Upon the applied reductive amination of GO, the O 1*s* peak at a binding energy (*BE*) of 532.5 eV is substantially reduced, while the N 1*s* signal centered at a *BE* = 400.1 eV appears, implying the successful removal of the oxygen groups and their partial substitution with nitrogen-containing ones. According to the quantitative analysis of the spectra prior to and after the applied treatment, the atomic concentration of oxygen dropped down by almost 10 times, from 46.58 at.% to 5.74 at.%, whereas the concentration of the introduced nitrogen was estimated to be 5.86 at.%.

To further assess the composition of the introduced nitrogen groups, the N 1*s* spectrum of the rGO-Am layer was examined after the deconvolution (Figure 1b). Four peaks were discerned centered at *BE*s of 398.7 eV, 400.1 eV, 401.3 eV and 403.5 eV, which matured from the presence of pyridines, amines, embedded graphitic nitrogen and pyridine-*N*-oxide moieties, respectively [33,34]. The relative concentrations of these nitrogen groups estimated by comparing the relative integral intensity are calculated to be 17.34%, 74.47%, 5.52% and 2.67%, respectively. Thus, the atomic concentration of the amine groups in the synthesized rGO-Am is ca. 4.36 at.%, which corresponds to the highest reported values in terms of the direct covalent grafting of the graphene layer without any linker [34,35,36].

Given the XPS data on the composition of the functional groups and the size distribution of the rGO-Am derived from the measurements using the LD technique (Supporting Information, Figure S1), the molar ratio of the introduced amines was estimated. The calculated value was ca. 2.57 mmol/g. Details on the calculation of the molar ratio are presented in Supporting Information, Section S2.

Moving from rGO-Am to Am-ABd, the successful immobilization of the monoclonal antibodies is indicated by a drastic rise in the O 1*s* and N 1*s* signals in the survey spectrum, complemented by the appearance of the S 2*p* peak centered at a *BE* = 163.5 eV [37]. This peak matures from the S atoms of some amino acids, which constitute the antibodies, as thiol components are predominantly presented in the high-resolution S 2*p* core-level spectra after their deconvolution (Supporting Information, Figure S2). The quantitative analysis yields atomic concentrations of 72.58 at.%, 15.27 at.%, 9.30 at.% and 2.85 at.% for the C, O, N and S, respectively. Unfortunately, the precise molecular structure of the immobilized monoclonal antibodies is unknown, and the immobilization efficiency cannot be estimated from these data. However, the predominant role of the performed amination in the immobilization of the antibodies is pointed out by the comparative analysis of the Am-ABd survey spectra with one of the pristine rGO treated by the same grafting procedure (Supporting Information, Figure S3). No signs of the N 1*s* and S 2*p* core-level lines are indicated in the rGO survey spectrum, and the intensity of the O 2*s* signal is negligible, signifying all the antibodies were removed by the applied purification procedure.

Furthermore, no positive effect of the addition of the NHS and sulfo-NHS into the reaction mixture was shown. The use of these reagents is known to commonly facilitate the conjugation of biomolecules [38,39,40]. However, in our case, no enhancement in the degree of antibody grafting was found from the comparative analysis of the survey spectra acquired for the samples derived in otherwise identical conditions (Supporting Information, Figure S4) without or with the addition of NHS and sulfo-NHS.

The introduction of a high number of antibodies to the surface of the rGO-Am layer is further reflected by the changes in the N 1*s* and C 1*s* high-resolution spectra. In the N 1*s* spectrum, the peak of the amines becomes dominant with its relative intensity rising to 93.41% due to the contribution of amines from the amino acids of the antibodies. Even more drastic changes are seen from the C 1*s* spectra presented in Figure 1c. Upon the immobilization procedure, the oxygen-related peaks centered at *BE*s of 286.9 eV, 288.2 eV and 289.1 eV increase, originating from the presence of the C-OH&C-O-C, C=O and COOH groups, respectively [33,41,42], and completely absent in the rGO-Am C 1*s* spectrum due to the elimination of these oxygen moieties during amination. The concentration of these oxygen groups enhances from 4.23 at.%, 0.97 at.% and 1.48 at.% to 24.90 at.%, 12.70 at.% and 1.5 at.%, respectively, as seen from the quantitative data derived from processing the deconvoluted C 1*s* spectra and summarized in Table 1.

Furthermore, the C-N peak centered at a *BE* = 285.9 eV and related to the presence of nitrogen-containing groups also rises with the increase in the corresponding relative concentration from 5.85 at.% to 13.45 at.%. Conversely, the asymmetric C=C peak with a *BE* of 284.7 eV related to the carbon atoms of the conjugated π-bonding network and dominant in the rGO-Am spectrum becomes less prominent, whereas the C-C peak with a *BE* of 285.1 eV related to the non-conjugated carbon atoms of the amino acid backbones rises [43,44]. Notably, no signs of the C-V peak centered at a *BE* = 283.9 eV presented in the initial GO, is seen in both rGO-Am and Am-ABd spectra. This spectral feature originates from the presence of non-terminated carbon atoms at the edges of vacancies due to steric limitations. Thus, its disappearance upon the performed treatment implies that all the carbon atoms are either functionalized or contribute to the π-conjugated network [44]. 

**Figure 2:**
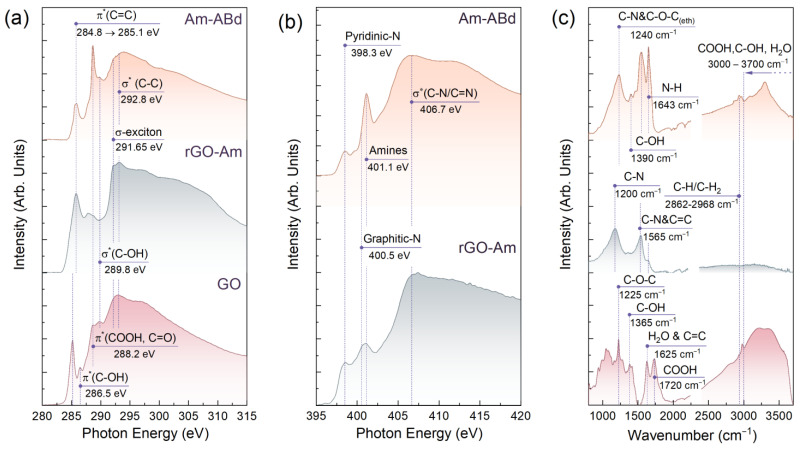
Characterization of the CMGs’ chemistry via X-ray absorption and ATR-FTIR spectroscopies. (**a**) C *K*-edge X-ray absorption spectra; (**b**) N *K*-edge X-ray absorption spectra; and (**c**) FTIR-ATR spectra of the studied materials. The absorption bands at 930–1080 cm^−1^ in the GO spectrum are not denoted. The CO_2_ signal at *v* = 2220–2400 cm^−1^ is cut out for clarity.

The XPS data are well supported by the results of the XAS studies. Figure 2a displays the C *K*-edge X-ray absorption spectra of the studied materials. The reductive amination of the GO resulted in the vanishing of a set of resonances contributed by the C 1*s*-π* or C 1*s*-σ* electron transitions in the edge-located C-OH, basal-plane C-OH and C=O/COOH groups and centered at *hv* = 286.5 eV, *hv* = 289.8 eV and *hv* = 288.2 eV, respectively [42,45,46]. In turn, the C 1*s*-π* resonance at *hv* = 284.8 eV, which matures from the electron transitions in the C = C bonds at pristine sp^2^-domains, shifts towards *hv* = 285.1 eV with an increase in its ratio to the σ*-resonance of the graphene lattice (C 1*s*-σ* peak) from 0.57 to 0.78. Complemented by the appearance of the σ*-exciton resonance centered at *hv* = 291.65 eV, these transformations in the X-ray absorption spectrum indicate the recuperation of the extended π-conjugated graphene network upon amination [34,47]. At the same time, the subsequent immobilization of the antibodies results in a drastic reduction in the C 1*s*-π* (C=C) resonance intensity, the vanishing of the σ*-exciton resonance and the simultaneous increase in the C 1*s*-π* (C=O, COOH) resonance owing to the introduction of a high number of aliphatic carbon as well as carbonyl and carboxyl groups from the grafted antibodies. The N *K*-edge X-ray absorption spectra of the rGO-Am and Am-ABd layers exhibited in Figure 2b also demonstrate the enhancement of the spectral features related to the moieties present in the amino acids. Notably, the N 1*s*-π* resonance of the amine groups centered at *hv* = 401.1 eV and initially presented in the rGO-Am spectra is drastically enhanced, showing an increase in the number of amines [34].

The ATR-FTIR spectra displayed in Figure 2c finally justify the asserted conversion of GO into the aminated graphene and its subsequent grafting by the monoclonal antibodies. Upon the applied amination, the large set of absorption bands at *v* = 935 cm^−1^, *v* = 1040 cm^−1^, *v* = 1225 cm^−1^, *v* = 1365 cm^−1^ and *v* = 1720 cm^−1^ related to lactols, edge-located hydroxyls, epoxides, basal-plane hydroxyls and carboxyls along with a broad hump at *v* = 3000–3700 cm^−1^ contributed by the overlapping absorption bands of the C-OH, H_2_O and COOH groups disappears or substantially diminishes [48]. In turn, the absorption bands at *v* = 1200 cm^−1^, *v* = 1565 cm^−1^ and *v* = 1643 cm^−1^ related to the C-N bending and N-H deformation modes increase in the rGO-Am spectrum [49], becoming the dominant ones. Notably, no N-H stretching modes commonly appearing in the range of *v* = 3000–3350 cm^−1^ are indicated, probably due to the quenching effect of the graphene layer, although this feature requires additional studies.

The subsequent introduction of the antibodies leads to the further modification of the ATR-FTIR spectrum with an enhancement in the already presented absorption bands, originating from the amine groups along with the appearance of a set of new ones. Particularly, the C-N peak initially present at *v* = 1200 cm^−1^ shifts towards higher wavenumbers due to being mixed with the band appearing at *v* = 1280 cm^−1^ maturing from the C=O bonds. Meanwhile, the C-OH band centered at *v* = 1390 cm^−1^, the C-H/C-H_2_ ones at *v* = 2862–2968 cm^−1^ and a broad peak from the N-H stretching at *v* = 3298 cm^−1^ appear [49,50]. Considering these bands almost perfectly fit the spectra of the monoclonal antibodies measured prior to the immobilization procedure (Supporting Information, Figure S5), such a modification of the rGO-Am spectrum after its treatment points out the successful covalent grafting of this graphene derivative by antibodies, completing the XPS and XAS data.

### 3.2. Morphology of the rGO-Am Prior to and after the Immobilization of Antibodies

**Figure 3:**
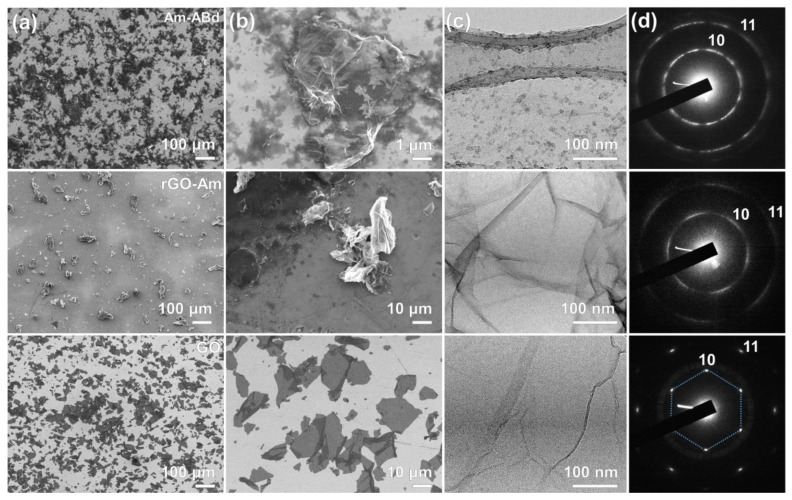
Morphology of the derived CMGs. (**a**,**b**) SEM images; (**c**) TEM images; and (**d**) corresponding ED patterns of the initial GO (**bottom row**), rGO-Am (**middle row**) and Am-ABd (**upper row**) layers.

The alterations in the materials’ chemistry upon the GO conversion in the aminated graphene and subsequent attachment of antibodies are accompanied by changes in their morphology, as reflected by further microscopic studies. Figure 3a,b display low- and high-magnification SEM images of the studied materials, respectively, signifying initially lamellar and flat GO flakes that become crumpled with a high number of folds of up to several micrometers in height. Such an extensive corrugation of the graphene layer upon the amination is related to the elimination of the basal-plane oxygen groups, the flattening the graphene layer due to the electrostatic repulsion and the graphene lattice distortion induced by the amine groups [51,52]. Tending to form clusters in rows [53], amines promote the formation of folds to compensate for the induced strain, resulting in the crumpled structure of this graphene derivative. Additionally, the possible interaction between the amines and the retained oxygen groups, particularly the carboxyl ones, also can contribute to the rolling of the graphene layers. It is worth noting that rGO-Am exhibits a corrugated structure regardless of the solvent and substrate type as well as the changes in the parameters of the employed deposition process, implying this is an inherent feature of this material.

Crumpling is also indicated by the acquired TEM image displayed in Figure 3c. Despite several folds also present in the GO layer, their extension and scale are substantially enhanced in the case of rGO-Am, as seen from the image and even more clearly by comparing the ED patterns of GO and rGO-Am, which are exhibited in Figure 3d. The ED pattern of the former is presented by a single set of six sharp (10) and (11) diffraction maxima, which become substantially blurred upon moving to the rGO-Am, which arises from the deviations in the angle between the electron beam and the graphene surface due to the folds [54,55].

Contrary to the pristine rGO-Am, its counterpart with the immobilized antibodies demonstrates a more lamellar structure, as shown by both SEM and TEM imaging. Such a backward change in the morphology derives from the appearance of antibodies on the surface of the rGO-Am layer. Particularly, the distance between antibodies estimated from the TEM imaging is about 10–20 nm, although their shape is not well defined on the corresponding images. Owing to such a dense distribution of antibodies, any considerable distortion in the rGO-Am layer is restricted due to steric limitations. Notably, despite the flattening of the rGO-Am layer, the ED pattern is found to further transform into a ring shape due to the appearance of a diffraction signal from the aliphatic carbon from the antibodies. Nevertheless, the indicated partial restoration of the rGO-Am lamellar structure upon it grafting by antibodies is advantageous for the formation of continuous uniform films in terms of the fabrication of the sensing layers for the biosensing platforms.

### 3.3. Electronic Structure of the rGO-Am and Am-ABd Layers

**Figure 4:**
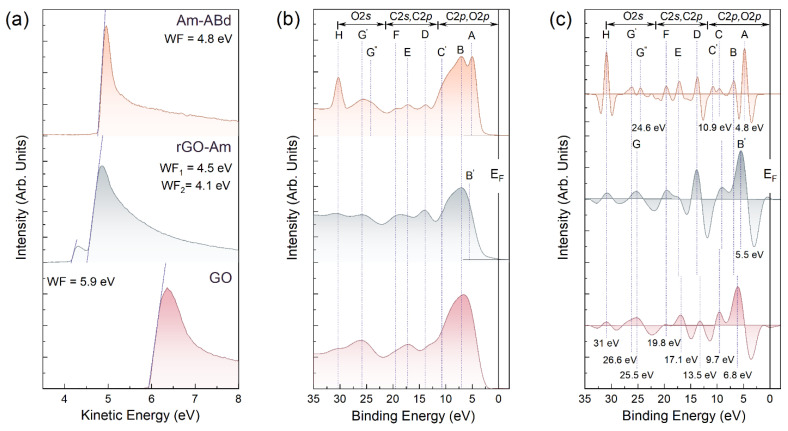
(**a**) SE cut-off spectra, (**b**) VB photoemission spectra and (**c**) corresponding second derivatives (-d^2^I/dE^2^) of the materials under study.

The WF values and the VB structure were further examined to assess the effect of the amination and grafting by antibodies on the electronic structure of the graphene layer. Figure 4a displays the SE cut-off spectra of the studied materials, allowing us to estimate their WF and, correspondingly, the electron-donating or electron-withdrawing effect of the presented functional groups. The WF value of GO is 5.9 eV, which coincides with that of the published data on this graphene derivative [56,57]. In turn, the performed amination resulted in a shift in the slope of the SE cut-off spectra towards lower kinetic energies along with the appearance of an additional peak, one-fifteenth of the intensity of the main one. The corresponding WF values are estimated to be 4.5 eV and 4.1 eV. The former is related to the WF of the areas of the pristine π-conjugated graphene network restored upon the elimination of oxygen groups, whereas the latter is asserted to originate from the local areas of a high concentration of amine groups owing to their electron-donating effect [51,57].

Conversely, this feature disappears after the immobilization of the antibodies, which is complemented by a slight shift in the SE cut-off spectrum to higher energies with a corresponding WF value of 4.8 eV, which is 0.3 higher than that of pristine rGO. The former effect additionally supports the covalent bonding of the antibodies, since the electron-donating effect from these moieties completely disappears. Meanwhile, the increase in the WF value implies a considerable electron-withdrawing effect of the grafted antibodies, which is also reflected by the narrowing of the SE spectra near the cut-off threshold.

The strong effect of amination and grafting by the antibodies on the electronic structure of the graphene layer is further evidenced by the examination of the VB spectra and their second derivatives taken with the opposite sign, which are displayed in Figure 4b,c. The use of second derivatives makes it possible to unveil broadened, overlapping spectral features and to precisely define their positions, which is of high interest in terms of inspecting the appearance of local electronic states induced by the introduced functionalities.

Upon amination, the density of states (DOS) in the region of 5–13 eV, stemming from both the C 2*p* electrons of the graphene network and the O 2*p* electrons of the oxygen groups, diminishes along with the vanishing of the *G* band at a *BE* = 26.2 eV, which is related to the electronic states from the O 2*s* atomic orbitals. Complemented by the appearance of discernable D, E, and F bands of the $K_{1}^{+}\left(\sigma_{3}\right),$ converged $K_{3}^{+}\left(\sigma_{3}\right),$ $M_{1u}^{+}\left(\sigma_{2}\right)$ and $M_{1g}^{+}(\sigma_{1})$ states of four high-symmetry points of the graphene Brillouin zone [58,59,60], these alterations in the valence band structure signify an almost complete elimination of the oxygen groups in accordance with the spectroscopic data. Accordingly, the DOS near the Fermi level appears due to the recuperation of the graphene π-conjugated network and the appearance of the *B*’ band, attributed to the more pronounced DOS related to the 2p-σ electron states of the graphene lattice.

At the same time, no additional electronic states that might be related to the presence of amines can be distinguished, unlike the previously demonstrated introduction of a set of localized states related to the molecular orbitals (*Mo*s) of the introduced carboxyl and ketone groups in C-xy and carbonylated (C-ny) graphenes [43,61,62]. In turn, these states reveal themselves in the VB spectra of the Am graphene after the immobilization of antibodies. Particularly, a sharp *A* peak centered at 4.8 eV along with a less pronounced but discernable one on the second derivative spectrum *B*, *C* and *C*′ spectral bands at *BE*s of 6.8 eV, 9.7 eV and 10.9 eV, respectively, are indicated. As was demonstrated for the C-xy and C-ny graphene in Ref. [40] in accordance with the earlier studies on formaldehyde and formic acid [63,64], the *A* and *B* bands arise from the *MO*s occupied by oxygen 2p nonbonding lone pair electrons n’O2*_p_* (HOMO) of the ketone and carboxyl groups, respectively. Conversely, the *C* and *C*′ bands correspond to localized states of the σ(C-O) and π(O-C-O) bonds in these oxygen functionalities. Furthermore, the depletion of the DoS with two maxima *G*′ and *G*″ with BEs of 24.6 eV and 26.2 eV, respectively, is observed, originating from the enhancement in the relative contribution of *MO*-related electronic states of the σ(C-O) bonds in the hydroxyl (*G*′) and ketone (*G*″) constituents of the amino acids. The *H* peak at a *BE* = 31.0 eV, dominating in the VB spectra of Am-ABd, corresponds to the Na 2*p* core-level line of sodium, which inevitably remains at low concentrations physiosorbed on CMGs upon their synthesis.

Besides the appearance of the *MO*-related electronic states, the immobilization of antibodies is also found to introduce a band gap of ca. 0.6–0.8 eV. We attribute this to the strong electron-withdrawing effect of the bonded antibodies already indicated by the aforementioned examination of the materials’ WF, since the graphene-related DOS maxima *D*, *E* and *F* are retained in the VB spectra, implying the absence of any graphene network distortion. Given the high intensity of the *A* and *B* bands, no interaction between the ketones and carboxyls of the antibodies with the graphene layer is asserted, as the hydrogen bonding results in the diminishing of the DOS related to the lone pair electrons n’O2*_p_*, that is, the *A* and *B* bands [65]. This implies antibodies exhibit a free-standing configuration on the Am surface after immobilization, being active for the conjugation with the target biomarker with a simultaneous strong effect on the charge transport in the graphene layer, both of which are advantageous for the application of Am-ABd layers in biosensing.

### 3.4. Sensing Performance of the Biosensing Chips Composed of Am-ABd Layers

**Figure 5:**
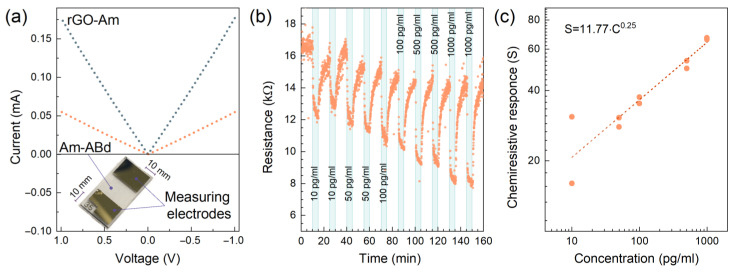
Sensing performance of the fabricated chip. (**a**) *I*–*V* curves for the rGO-Am and Am-ABd layers taken at both d.c. electric field directions under dry air conditions; the insert shows the photo of the fabricated biosensing chip. (**b**) The resistance transient recorded under exposure to PBS solution with IgM immunoglobulins of various concentrations in the range of 10–1000 pg/mL. (**c**) The dependence of chemiresistive response on the concentration of the IgM immunoglobulins.

Given the acquired data on the chemistry, morphology and electronic structure of the Am layer after its covalent grafting, the acquired modified CMG was trialed as a sensing layer for a chemiresistive biosensing platform. Figure 5a displays the schematic model of the fabricated two-electrode chemiresistive biosensor and the *I–V* curve taken at both d.c. electric field directions under dry air conditions. The linear character of the *I–V* curve shows good Ohmic contact between the Am-ABd film and electrodes, ensuring the absence of significant potential barriers at the contact that could affect the performed measurements. The resistance of the Am-ABd layer is estimated to be ca. 18.194 kΩ, whereas the resistance of the initial rGO-Am prior to the immobilization was ca. 5.649 kΩ, almost three times lower. These results evidence the decrease in the charge transport upon the immobilization of antibodies due to the stated electron-withdrawing effect and reduction in the DOS near the Fermi level.

Figure 5b displays a typical *R*(*t*) transient recorded upon the consecutive exposition towards the PBS solution and mix of the PBS solution with IgM immunoglobulins from human serum with concentrations of 10, 50, 100, 500 and 1000 pg/mL. For each concentration, the sensor was exposed for 10 min. It was twice interrupted by the purifying the surface of biosensors with 10 min of treatment with a GHCl detergent and with 10 min of treatment with the stock PBS solution.

The reproducible reduction in the resistance of the Am-Abd layer upon the deposition of PBS solution with IgM immunoglobulins is indicated, with the magnitude of chemiresistive signal (*S*) following the concentration of the analyte, except for the first exposure, for which the signal is even higher than that for the following higher concentrations. This originates from the retention in part of the immunoglobulins in the localized areas of the film upon the first deposition, occupying the potential adsorption sites and, thus, reducing the chemiresistive signal for the following exposures. Nevertheless, the resistance increases to almost the initial values after the treatment by the GHCl detergent and PBS solution, showing the applicability of the fabricated prototype in carrying out multiple measurements. Furthermore, the dependence of the chemiresistive response on the concentration S(C) is found to correspond to Freundlich’s isotherm of the 𝑆 = A∙𝐶^α^ type, for which the factor and power coefficient (A and α) are estimated to be 11.77 and 0.25, respectively. The magnitude of *S* towards the 10 pg/mL is ca. 16%, whereas this value increases up to ca. 66% when moving to IgM concentrations of 1000 pg/mL. Accordingly, the sensitivity coefficient calculated as *S/C* yields a value of 1.6%/pg∙mL^−1^ at low concentrations of immunoglobulins with a reduction at higher concentrations of 1000 pg/mL to 0.07%/pg∙mL^−1^, owing to the progressive reduction in the number of the antibodies accessible for binding. Yet, the calculated chemiresistive response is among the highest ones reported in the literature, assuming comparable sensitivity characteristics of the fabricated prototype with state-of-the-art devices [20,22,25,66,67,68,69], as summarized in the Supporting Information, Table S1.

To further assess the selectivity of the fabricated prototype, additional measurements while exposing the Am-ABd layer towards BSA instead of the IgM immunoglobulins were carried out (Supporting Information, Figure S6). Additionally, the chemiresistive response of the pristine Am layer without antibodies while being exposed to the IgM immunoglobulins was tested to determine the role of the immobilized antibodies in the specified sensing performance of the Am-ABd layer (Supporting Information, Figure S7). As seen for the displayed *R*(*t*) graphs, no pronounced chemiresistive response was indicated, which signifies both the high selectivity of the fabricated prototype and the predominant role of the immobilized antibodies in the detection of the target analyte.

## 4. Conclusions

Thus, we emphasize the advantageous application of aminated graphene for the covalent immobilization of monoclonal antibodies towards human IgM immunoglobulins as a modeling biorecognizing agent. The introduced amines play a predominant role for this purpose in terms of acting as anchoring sites for the antibodies with the formation of covalent amide bonds between the graphene and the Fc domains of the antibodies. Without the amines presented, no stable immobilization of the antibodies on the graphene layer is observed.

Both the introduction of amines and the subsequent grafting of antibodies are revealed to substantially yet differently affect the morphology and electronic properties of the graphene layer. While the introduction of amines is found to induce the corrugation of the graphene flakes, the subsequent introduction of antibodies is demonstrated to compensate for this effect with a partial restoration of the lamellar structure. This matures from the achieved uniform dense distribution of the immobilized antibodies, inducing stering limitations for the corrugation of the graphene layer and, thus, smoothing it. 

A substantial alteration of the VB structure is revealed upon the immobilization of antibodies, as reflected by the appearance of a band gap and the rise in a set of localized *MO*-related electronic states, originating from the energy levels of the C-O bonds in the oxygen moieties of the amino acids. Furthermore, a strong electron-withdrawing effect of the immobilized antibodies is revealed, which exceeds the electron-donating effect of the amine groups and compensates for the local reduction in the WF value induced by these functionalities.

The high performance of the acquired material for biosensing has been shown. A high sensitivity and selectivity for detecting the target analyte are demonstrated for the fabricated biosensor prototype with a limit of detection as low as 10 pg/mL as compared to that of state-of-the-art biosensing devices.

Taken together, these findings advance and outline graphene derivatives’ application in biosensing as well as hint at how the features of graphene morphology and physics are altered upon their functionalization and further covalent grafting by biomolecules.

## Figures and Tables

**Table 1:** Relative concentrations of the functional groups in the examined CMGs.

Component	C-V	C=C	C-C	C-OH&C-O-C	C=O	COOH	C-N	C/O Ratio
Binding Energy [eV]	284.6	286.8	287.0	288.2	288.9			
GO	3.97	55.34	4.23	29.70	4.95	1.81	-	2.61
rGO-Am	1.79	80.35	5.33	4.23	0.97	1.48	5.85	12.25
Am-ABd	0.00	28.50	18.95	24.90	12.70	1.50	13.45	2.46

## Data Availability

The data presented in this study are available on request from the first author.

## Supplementary Materials

The following supporting information can be downloaded at: https://www.mdpi.com/article/10.3390/nano13111730/s1. Figure S1: Size distribution of the rGO-Am flakes in the Isopropyl suspension measured by means of the Laser Diffraction method; Figure S2: High-resolution S 2*p* spectrum of Am-ABd after the deconvolution procedure; Figure S3: Survey spectra of the rGO-Am and pristine rGO after the immobilization of the antibodies (Am-ABd and rGO-ABd, respectively) carried out in otherwise identical conditions. No signs of the N 1*s* and S 2*p* signals can be indicated in the rGO-Abd spectrum, assuming the absence of the immobilized antibodies retained after the purification procedure; Figure S4: Survey spectra of the rGO-Am grafted by the antibodies without (left) and with the addition of either NHS (central) or Sulfo-NHS (right). Insets demonstrate the element composition of the acquired samples, pointing out lower immobilization efficiency in the case of employing both NHS and Sulfo-NHS; Figure S5: ATR-FTIR spectra of the monoclonal antibodies towards IgM immunoglobulins of human. The CO_2_ signal at v = 2220–2400 cm^-1^ is cut out for clearness; Table S1: Comparison of the fabricated prototype’s sensing performance to the published data; Figure S6: The resistance transient recorded for the Am-ABd layer under exposure to PBS solution with BSA of varied concentration in the range of 50–1000 pg/mL; Figure S7: The resistance transient recorded for the rGO-Am layer under exposure to PBS solution with immunoglobulins IgM of varied concentration in the range of 50–1000 pg/mL. References [70,71,72,73] are cited in the supplementary materials.
