# Supplementary material for: Graphene Amination towards Its Grafting by Antibodies for Biosensing Applications
Source: Nanomaterials (Basel). 2023 May 25;13(11):1730. doi: 10.3390/nano13111730 (PMC10254260; doi:10.3390/nano13111730)
Supplement: Supplementary file 1 [file nanomaterials-13-01730-s001.zip › nanomaterials-2400070-supplementary.pdf]

Supporting Information for:

Graphene Amination towards Its Grafting by Antibodies for Biosensing Applications

Maxim K. Rabchinskii [1,*], Nadezhda A. Besedina [2], Maria Brzhezinskaya [3], Dina Yu. Stolyarova [4], Sergei A. Ryzhkov [1], Sviatoslav D. Saveliev [1], Grigorii A. Antonov [1], Marina V. Baidakova [1], Sergei I. Pavlov [1], Demid A. Kirilenko [1], Aleksandr V. Shvidchenko [1], Polina D. Cherviakova [1], Pavel N. Brunkov [1]

[1] Ioffe Institute, Politekhnicheskaya St. 26, Saint Petersburg, 194021, Russia

[2] Department of Physics, Alferov University, 8/3 Khlopina Street, Saint-Petersburg, 194021, Russia

[3] Helmholtz-Zentrum Berlin für Materialien und Energie, Hahn-Meitner-Platz 1, 14109 Berlin, Germany

[4] NRC "Kurchatov Institute", Akademika Kurchatova pl. 1, Moscow, 123182, Russia

*Correspondence: rabchinskii@mail.ioffe.ru

Section S1. Laser Diffraction characterization of the rGO-Am

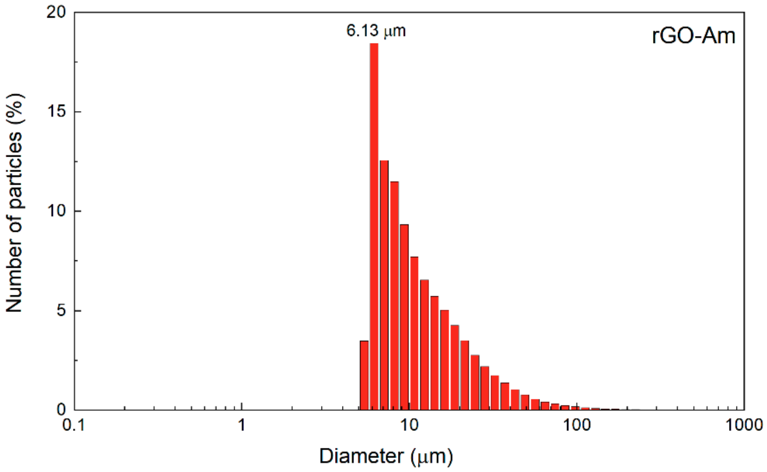

Figure S1. Size distribution of the rGO-Am flakes in the Isopropyl suspension measured by means of the Laser Diffraction method.

Section S2. Calculations of the molar ratio and number of functional groups in rGO-Am

To calculate the molar ratio of the amines as well as other oxygenic and nitrogen functionalities the following relation was used:

$$v = \frac{m_{rGO-Am} * N_A}{m_{Flake} * N_{group}} \quad (S1)$$

where m_{rGO-Am} is the total mass of the rGO-Am, N_A is the Avogadro constant, m_{Flake} is the mass of a single flake, and N_{group} is the total number of the functional groups, for which the molar ratio is calculated – amines in our case. To estimate the mol/g ratio, the m_{rGO-Am} is taken as 1 g, whereas the m_{Flake} and N_{group} are the calculated values. The former value is estimated from the following relation:

$$M_{flake} = M_{hex} * N_{hex} \quad (S2)$$

where M_{hex} is the mass of one hexagon, and N_{hex} is the number of hexagons that make up the flake. The mass of one hex can be calculated as follows:

$$M_{hex} = 6 * Da(C) * \frac{1}{3} + (\sum Da_{fg} * at.\%) \quad (S3)$$

where Da_{fg} is Dalton units of atoms of functional groups, also known as unified atomic mass unit, at.% - is the relative content of the functional groups determined from the processed C 1s and N 1s X-ray photoelectron spectra. The N_{hex} is calculated as follows:

$$N_{hex} = S_{fl}/S_{hex} \quad (S4)$$

The S_{fl} refers to the mean area of the flakes estimated from the size distribution determined experimentally using laser diffraction, whereas S_{hex} is calculated as:

$$S_{hex} = 1{,}5 * \sqrt{3} * a \quad (S5)$$

where a is the length of a single C=C bond in the graphene network, equal to 0.142 nm. In turn, the N_{group} can be estimated by the following relation:

$$N_{group} = N_{hex} * at.\% \quad (S6)$$

Given this relations, the atomic concentration of the functional groups from the XPS data (**Table 1**), the average diameter of rGO-Am flakes of 6.13 µm (**Supporting Section, S1**) and the $Da_{NH3} = 17$ the molar ratio of amines was calculated to be 2.57 mmol/g, whereas their number in 1 g of rGO_Am is estimated to be ca. $1.55*10^{21}$.

Section S3. Processing high-resolution S 2p spectrum of Am-ABd

Figure S2 displays the high-resolution S 2p spectrum of Am-ABd after its deconvolution. According to it, sulfur moieties in the Am-Abd are mainly featured by thiols or disulfides, revealing themselves by the presence of the C-S ($2p_{3/2}$) and C-S ($2p_{1/2}$) doublet centered at *BE*s of 163.7 eV and 164.9 eV, respectively [R1]. Besides, a minor contribution of the sulfur bonded to oxygen, such as sulfates or sulfites, is manifested by the presence of the discerned doublet of SO_x^{2-} ($2p_{3/2}$) and SO_x^{2-} ($2p_{1/2}$) with *BE*s of 168.2 eV and 170.0 eV, respectively [R2]. Given the relation between the areas of the corresponding doublets, the relative content of thiols or disulfides is estimated to be 81.2%, whereas the rest 18.8 % of sulfur moieties are oxidized ones.

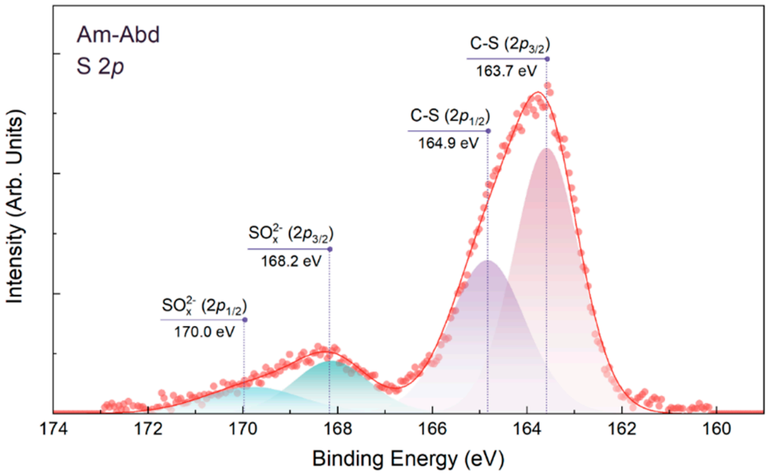

Figure S2. High-resolution S 2*p* spectrum of Am-ABd after the deconvolution procedure

Section S4. Comparing immobilization efficiency between rGO-Am and pristine rGO

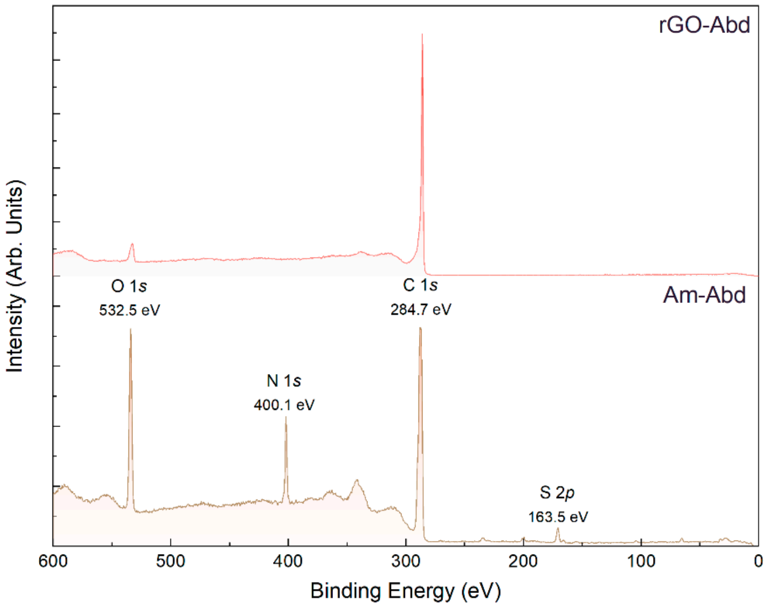

Figure S3. Survey spectra of the rGO-Am and pristine rGO after the immobilization of the antibodies (Am-ABd and rGO-ABd, respectively) carried out in otherwise identical conditions. No signs of the N 1*s* and S 2*p* signals can be indicated in the rGO-Abd spectrum, assuming the absence of the immobilized antibodies retained after the purification procedure.

Section S5. The effect of NHS and Sulfo-NHS on the immobilization efficiency

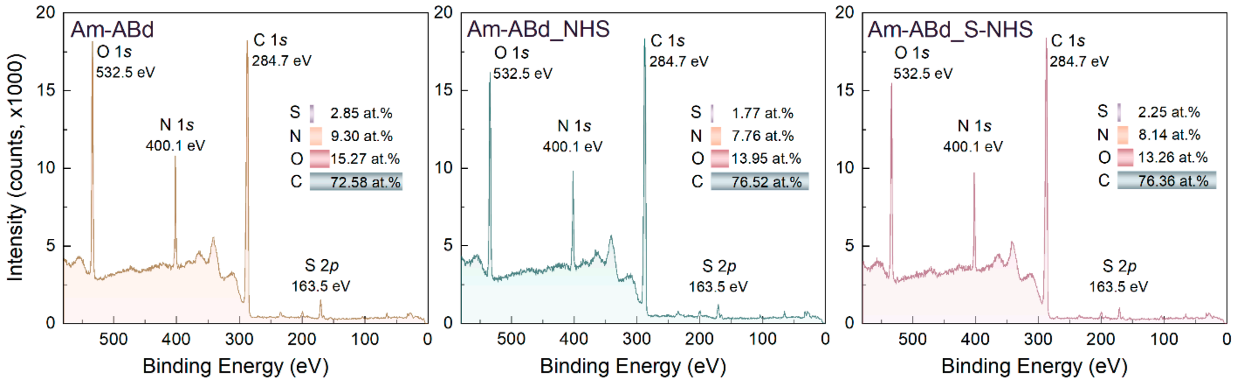

Figure S4. Survey spectra of the rGO-Am grafted by the antibodies without (left) and with the addition of either NHS (central) or Sulfo-NHS (right). Insets demonstrate the element composition of the acquired samples, pointing out lower immobilization efficiency in the case of employing both NHS and Sulfo-NHS.

Section S6. ATR-FTIR spectra of the monoclonal antibodies towards IgM immunoglobulins

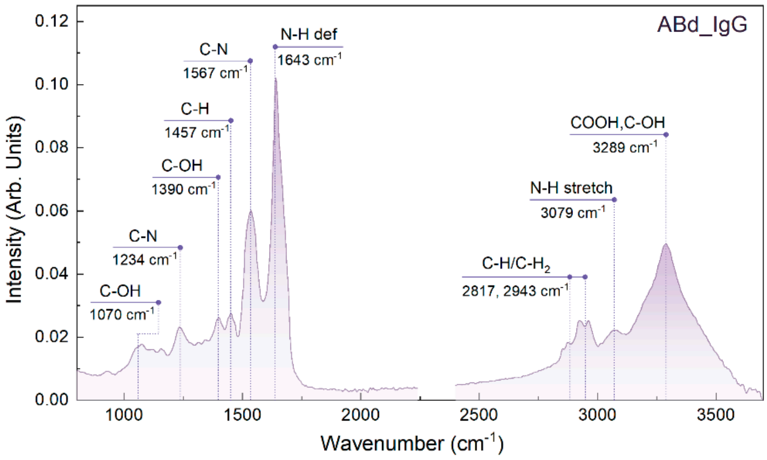

Figure S5. ATR-FTIR spectra of the monoclonal antibodies towards IgM immunoglobulins of human. The CO_2 signal at *v*=2220-2400 cm^{-1} is cut out for clearness.

Section S7. Comparison of the fabricated prototype's sensing performance to the published data.

Table S1. Comparison of the fabricated prototype's sensing performance to the published data.

No.	Sensing layer	Target	Shown LOD * (pg/ml)	Sensor Responsivity (%/pg/ml)	Selectivity proved	Ref.
1	Aminated graphene grafted with monoclonal antibodies	IgM immunoglobulins of human	10	1.64	Yes	This work
2	Carboxylated graphene modified with hemin-binding-aptamer (HBA)	Hemin	651.9	0.942	Yes	[R3]
3	Graphene layer with the methylene blue (MB) redox probe labeled aptamer	Aflatoxin B1	50	2.01	Yes	[R4]
4	Gold nanoparticles functionalized reduced graphene oxide grafted with ssDNA aptamer targeting HBsAg	HBsAg	0.125 fg/ml	7.86	Yes	[R5]
5	Carboxylated graphene grafted with the AO-01 aptamer against Hepatitis B protein	HBV capsid	10	0.433	No	[R6]
6	Graphene oxide and an aptamer of aflatoxin B1 covering anodized alumina membrane	aflatoxin B1	13	0.016	Yes	[R7]
7	graphene oxide nanosheet grafted by aptamer towards carcinoembryonic antigen	carcinoembryonic antigen in human serum	9.4	0.187	No	[R8]
8	Graphene layers grafted by DNA aptamers against the two β-LG variants A and B	β-lactoglobulin (β-LG) milk protein	20	0.004	Yes	[R9]
9	poly-3-amino-1,2,4-triazole-5-thiol/graphene oxide composite (P(ATT)-GO) and gold nanoparticles (AuNPs)	lipocalin-2 (LCN2) (neutrophil gelatinase-associated lipocalin)	300	15 min	Yes	[R10]
10	Aniline functionalized G/PANI nanodroplet-modified electrode	lipocalin-2 (LCN2)	21100	$6.7 \cdot 10^5$	No	[R11]

* - if not presented straightforwardly, the values of LoD and Sensor Responsivity in pg/ml and %/pg/ml are calculated from the data presented in the articles

Section S8. Chemiresistive response of Am-ABd layer towards BSA

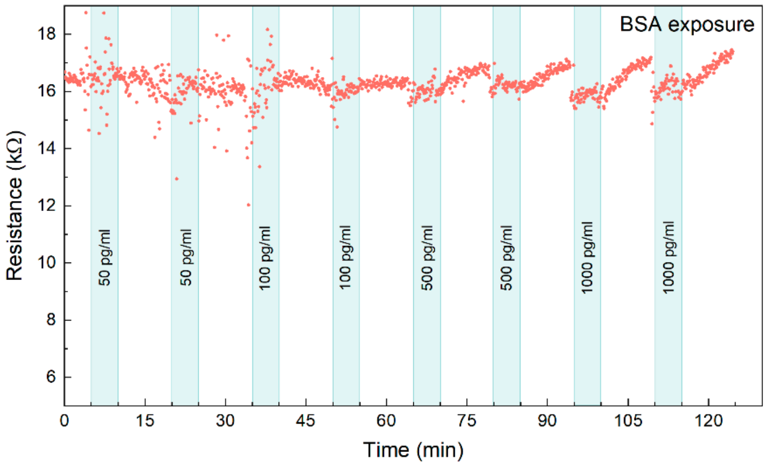

Figure S6. The resistance transient recorded for the Am-ABd layer under exposure to PBS solution with BSA of varied concentration in the range of 50-1000 pg/ml.

Section S9. Chemiresistive response of Am-ABd layer towards BSA

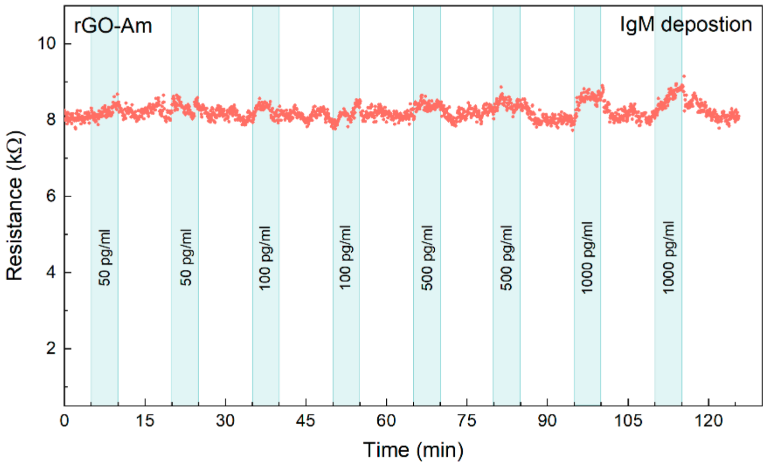

Figure S7. The resistance transient recorded for the rGO-Am layer under exposure to PBS solution with immunoglobulins IgM of varied concentration in the range of 50-1000 pg/ml

References:

[R1] Kannappan, S.; Yang, H.; Kaliyappan, K.; Manian, R.K.; Pandian, A.S.; Lee, Y.S.; Jang, J.-H.; Lu, W. Thiolated-graphene-based supercapacitors with high energy density and stable cycling performance. *Carbon* **2018**, *134*, 326-333. https://doi.org/10.1016/j.carbon.2018.02.036.

[R2] Wrobel, P. S.; Wlodarski, M. D.; Jedrzejewska, A.; Placek, K. M.; Szukiewicz, R.; Kotowicz, S.; Tokarska, K.; Quang, H. T.; Mendes, R. G.; Liu, Z. A comparative study on simple and practical chemical gas sensors from chemically modified graphene films. *Mater. Res. Express*, **2019**, *6*, 015607. https://doi.org/10.1088/2053-1591/aae6be.

[R3] Gao, L.; Xiao, Y.; Wang, Y.; Chen, X.; Zhou, B.; Yang, X. A Carboxylated Graphene and Aptamer Nanocomposite-Based Ap-tasensor for Sensitive and Specific Detection of Hemin. Talanta 2015, 132, 215–221. https://doi.org/10.1016/j.talanta.2014.09.010.

[R4] Goud, K. Y.; Hayat, A.; Catanante, G.; Satyanarayana, M.; Gobi, K. V.; Marty, J. L. An Electrochemical Aptasensor Based on Functionalized Graphene Oxide Assisted Electrocatalytic Signal Amplification of Methylene Blue for Aflatoxin B1 Detection. Electrochimica Acta 2017, 244, 96–103. https://doi.org/10.1016/j.electacta.2017.05.089.

[R5] Mohsin, D. H.; Mashkour, M. S.; Fatemi, F. Design of aptamer-based sensing platform using gold nanoparticles functionalized reduced graphene oxide for ultrasensitive detection of Hepatitis B virus. *Chem. Pap.* **2021**, *75*, 279–295. https://doi.org/10.1007/s11696-020-01292-1.

[R6] Rabchinskii, M. K.; Ryzhkov, S. A.; Besedina,N. A.; Brzhezinskaya, M.; Malkov, M. N.; Stolyarova, D. Yu.; Arutyunyan, A. F.; Struchkov, N. S.; Saveliev, S. D.; Diankin, I. D. et al. Guiding graphene derivatization for covalent immobilization of aptamers. *Carbon* **2022**, *196*, 264-279. https://doi.org/10.1016/j.carbon.2022.04.072.

[R7] Mo, R.; He, L.; Yan, X.; Su, T.; Zhou, C.; Wang, Z. et al. A novel aflatoxin B1 biosensor based on a porous anodized alumina membrane modified with graphene oxide and an aflatoxin B1 aptamer. *Electrochem Commun.* **2018**, *95*, 9–13. https://doi.org/10.1016/j.elecom.2018.08.012.

[R8] Hong, Z.; Chen, G.; Yu, S.; Huang, R.; Fan, C. A potentiometric aptasensor for carcinoembryonic antigen (CEA) on graphene oxide nanosheets using catalytic recycling of DNase I with signal amplification. *Anal Methods.* **2018**, *10*, 5364–71. https://doi.org/10.1039/C8AY02113A.

[R9] Eissa, S.; Zourob, M. In vitro selection of DNA aptamers targeting β-lactoglobulin and their integration in graphene-based biosensor for the detection of milk allergen. *Biosens Bioelectron*. **2017**, *91*, 169–74. https://doi.org/10.1016/j.bios.2016.12.020.

[R10] Tig, G. A.; Pekyardimci, S. An electrochemical sandwich-type aptasensor for determination of lipocalin-2 based on graphene oxide/polymer composite and gold nanoparticles. *Talanta* **2020**, *210*, 120666. https://doi.org/10.1016/j.talanta.2019.120666.

[R11] Yukir, J.; Wongtangprasert, T.; Rangkupan, R.; Chailapakul, O.; Pisitkun, T.; Rodthongkum, N. Label-free immunosensor based on graphene/polyaniline nanocomposite for neutrophil gelatinase-associated lipocalin detection. *Biosens. Bioelectron.* **2017**, *87*, 249-255. https://doi.org/10.1016/j.bios.2016.08.062.
